# Algal optics

**DOI:** 10.1103/fp9l-zykg

**Published:** 2026-02-01

**Authors:** Ming Yang, Sumit Kumar Birwa, Raymond E. Goldstein

**Affiliations:** Department of Applied Mathematics and Theoretical Physics, Centre for Mathematical Sciences, University of Cambridge, Wilberforce Road, Cambridge CB3 0WA, United Kingdom

## Abstract

Nearly a decade ago, it was discovered that the spherical cell body of the alga *Chlamydomonas reinhardtii* can act as a lens to concentrate incoming light onto the cell’s membrane-bound photoreceptor and thereby affect phototaxis. Since many nearly transparent cells in marine environments have complex, often nonaxisymmetric shapes, this observation raises fundamental, yet little-explored questions in biological optics about light refraction by the bodies of microorganisms. There are two distinct contexts for such questions: the *absorption* problem for *incoming* light, typified by photosynthetic activity taking place in the chloroplasts of green algae, and the *emission* problem for *outgoing* light, where the paradigm is bioluminescence emitted from scintillons within dinoflagellates. Here we examine both of these aspects of “algal optics” in the special case where the absorption or emission is localized in structures that are small relative to the overall organism size, taking into account both refraction and reflections at the cell-water boundary. Analytical and numerical results are developed for the distribution of light intensities inside and outside the body, and we establish certain duality relationships that connect the incoming and outgoing problems. For strongly nonspherical shapes, we find lensing effects that may have implications for photosynthetic activity and for the angular distribution of light emitted during bioluminescent flashes.

## I Introduction

**Fig. 1:**
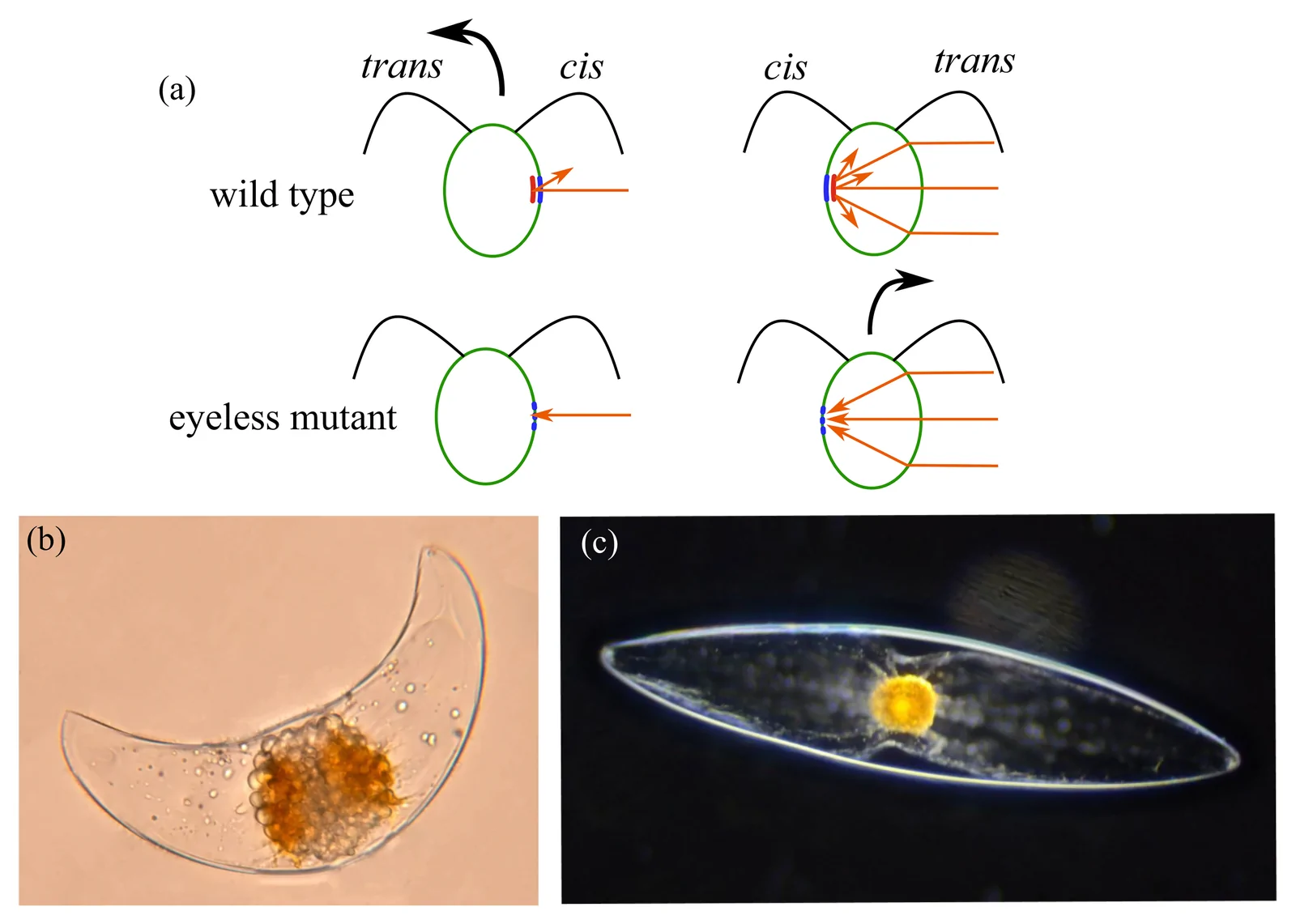
Algal optics. (a) Photoreceptor shading in wild-type and eyeless mutants of *Chlamydomonas reinhardtii*, after [2]. Dinoflagellates: (b) crescent-shaped *Pyrocystis lunula* and (c) spindle-shaped *Pyrocystis fusiformis* [4].

In a remarkably prescient paper [1], Kessler *et al*. found in 2015 that the spheroidal cell bodies of various green algae can act as lenses, bringing light from a distant source to a focus outside the cell. They suggested there might be functional significance to such a lensing effect and, indeed, in the following year, Ueki, *et al*. [2] found the first example, using the green alga *Chlamydomonas reinhardtii*. Phototaxis in *Chlamydomonas* is achieved through the coupling between signals received by a photosensor [3] and the beating dynamics of the two flagella that are anchored below the cell wall near the anterior pole of the cell. The photosensor [Fig. 1(a)] sits within a membrane at the periphery of the cell, and in wild-type cells has behind it an “eyespot,” a pigmented protein layer visible in bright field microscopy. Acting as a kind of “blind” or, more precisely, a quarter-wave plate [5], the eyespot reflects incident light; light passing through the cell from the opposite side of the eyespot is prevented from reaching the photosensor, while light incident on the photoreceptor from in front of the cell makes a double pass through the sensor. It is precisely this directionality that underlies the ability of cells to steer toward or away from light [6,7]. For example, in negatively phototactic mutants lacking the eyespot, light from behind the cell falls on the photosensor and one might naively expect the cell to be unable to perform phototaxis because of the isotropic detection of light. Yet, because of the lensing effect of the cell, the intensity of light falling on the photoreceptor from behind is greater than that from the forward direction, giving rise to a distinguishable signal and thus to net phototactic motion (erroneously) toward the light. It is thus plausible that the eyespot was selected by evolution precisely because, in providing directionality to the sensing of light, it improves the phototaxis of cells [8].

Lenses and lenslike properties of cells are known in other systems. For example, there is evidence for a lens *within* the eyespot of the dinoflagellate *Nematodinium* [9] that helps to gather light onto its photoreceptor. At the same time as the study of Ueki, *et al*., separate work on cyanobacteria [10] showed that its cell body can act as a lens, creating an uneven distribution of light intensity in the inner cell wall. Further work showed that a type of supramolecular machine for cell mobility is activated by that directional light [11]. These kinds of effects are now broadly understood for prokaryotes [12]. Finally, we note the case of diatoms, algae whose surface has a complex silica microstructure patterned on the scale of the wavelength of visible light. These structures induce wavelength-dependent optical properties [13,14] and diffraction [15].

Taking a broader view, there are important historical examples where lensing effects are thought to impact life’s processes, particularly with regard to plants [16]. For example, phototropism, the movement of plants toward light, is dependent on the fact that the cytoplasm of plant cells has a higher index of refraction than the surrounding medium (air or water). This was demonstrated by an experiment [17] that showed that this movement reverses direction when a plant is immersed in a medium with a higher index of refraction than the cytoplasm. Many investigations have focused on the effect of cell shape on photosynthesis in plants and fungi. For example, a study [18] of the filamentous fungus *Phycomyces blakesleeanus* showed that the light intensity at the cell surface is enhanced by a factor of ~2, while theoretical work suggests an even larger boost [19]. The epidermal cells of certain tropical plant species are believed to act as lenses, providing an advantage in light-gathering ability for shade plants [20], although later work found that the structure does not help to gather diffuse light [21].

The results summarized above indicate that lensing effects by the cell bodies of microorganisms can have a functional significance, even in the simplest possible geometry of a sphere. Yet, there are many freshwater and marine microorganisms with strikingly nonspheroidal shapes, as exemplified by dinoflagellates such as *Pyrocystis lunula* and *Pyrocystis fusiformis*, shown in Figs. 1(b) and 1(c). The properties of such complex, often nonaxisymmetric bodies remain largely unknown in the biological optics literature, although there is now a growing field of “freeform optics” [22–25] that considers nonaxisymmetric shapes. Motivated by these strange and wondrous forms, we commence an in-depth study of the field of “algal optics.”

Beyond their phototaxis, organisms such as green algae and dinoflagellates are photosynthetic and their chloroplasts, the organelles containing the photosynthetic apparatus, are in characteristic positions within the cell. In the case of dinoflagellates, the chloroplasts move around the cell in diurnal patterns [26–28], changing the absorption profile of the cytoplasm [29]. A natural question is whether lensing can enhance the intensity of light falling on chloroplasts. This is the “incoming” problem.

Dinoflagellates are among the many marine and fresh-water organisms that exhibit bioluminescence [30]. Unlike the steady glow of bioluminescent bacteria, these eukaryotes emit bright flashes of light in response to fluid or mechanical shear [31,32]. This light emanates from membrane-enclosed organelles termed “scintillons,” within which occur chemical reactions involving the protein luciferin. A second natural question is thus whether lensing can alter the spatial distribution of light emitted from such sources. This is the “outgoing” problem. An example of this is provided by very recent single-cell measurements on pennate diatoms showing that their elongated frustules channel internally generated chlorophyll fluorescence into strongly directional beams [33].

**Fig. 2:**
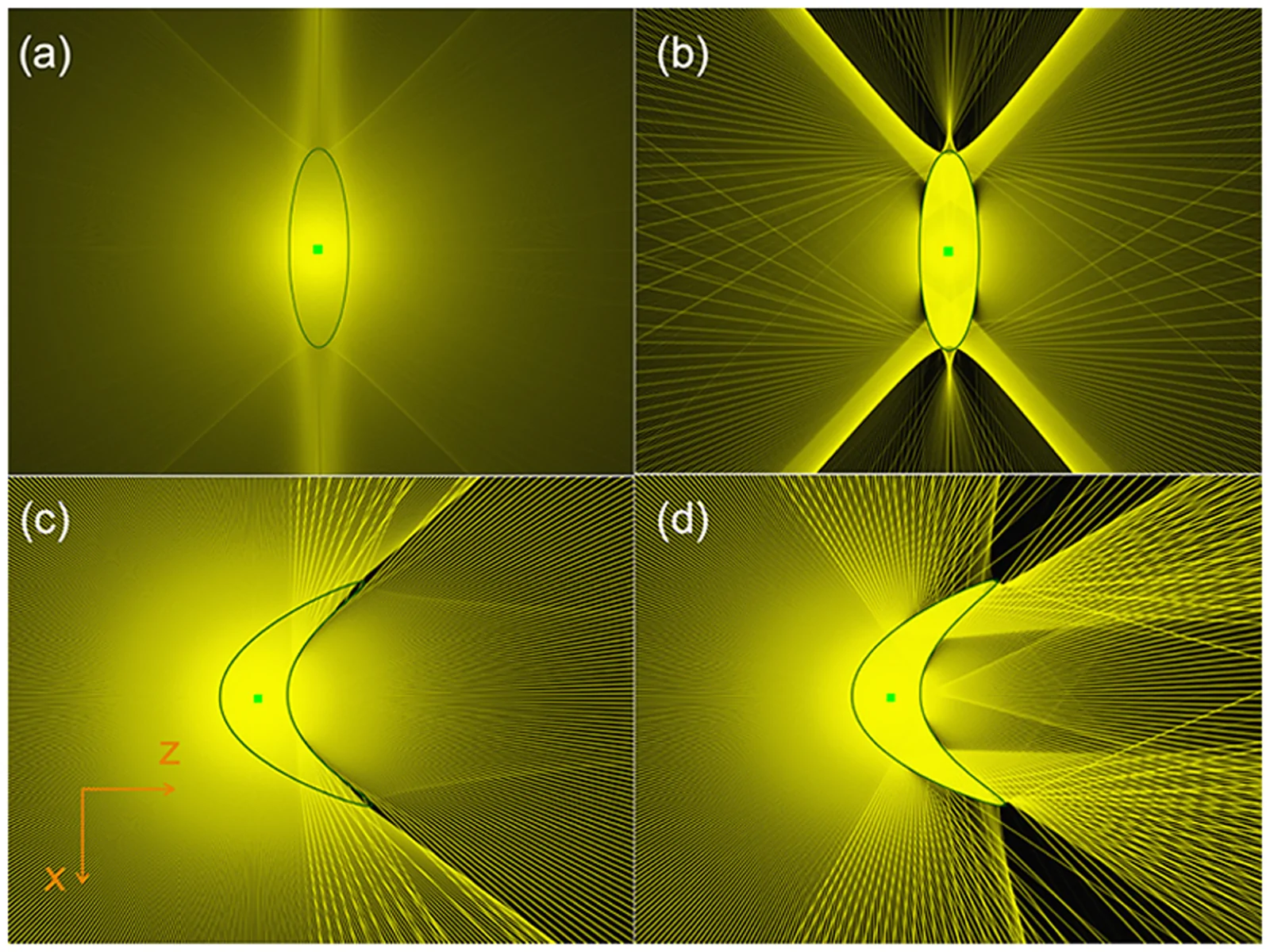
Ray-tracing simulations for dinoflagellate geometries [34] with a single central light source (green dot). Panels show two-dimensional (2D) slices (at *y* = 0) of the shapes of (a),(b) *P. fusiformis* $\left(z=\pm 0.3\sqrt{1-x^{2}}\right)$ and (c),(d) *P. lunula* $\left(z=x^{2}\pm 0.3\sqrt{1-x^{2}}\right)$, each at refractive indices of (a),(c) *n* = 1.1 and (b),(d) *n* = 1.5.

Figure 2 presents ray-tracing simulations from a ray optics simulator [34], illustrating an internal isotropic bioluminescent source positioned at the geometric center (green square) of two representative dinoflagellate cell shapes. The geometries are the spindle-shaped body of *P. fusiformis* [Figs. 2(a) and 2(b)] and the crescent-shaped body of *P. lunula* [Figs. 2(c) and 2(d)], each at two relative refractive indices *n*.

For the *fusiformis* geometry, the pattern of emission changes markedly with index. At *n* = 1.1 [Fig. 2(a)], rays preferentially exit along the long axis, producing a longitudinal beaming pattern directed toward the cell tips, similar to that observed in diatoms [33]. At *n* = 1.5 [Fig. 2(b)], stronger refraction redistributes energy laterally, enhancing escape along the short axis and producing a more axial pattern with side-directed beams.

For the *lunula* geometry, the dominant effect at low index is geometric asymmetry. At *n* = 1.1 [Fig. 2(c)], light preferentially escapes through the more open side of the crescent, generating a pronounced left-right intensity imbalance. At higher index [Fig. 2(d)], refraction and internal reflections reduce this imbalance, redistributing rays into broader exit angles and creating hotspots near the crescent tips. The left-right bias is still visible, but much less stark than at *n* = 1.1.

Together, these comparisons demonstrate how both cell shape and refractive index govern the angular distribution of light. Low-index cases emphasize geometry-driven leakage (longitudinal bias in *fusiformis*, strong left-right asymmetry in *lunula*), while higher-index contrast shifts the balance toward redistribution by refraction and internal reflection, producing lateral emission in *fusiformis* and tip-focused but more symmetric patterns in *lunula*.

In this paper, we study the incoming and outgoing problems with analytical and numerical methods. For the motivational cases of green algae and dinoflagellates, there is a reasonable separation between the size of the absorber or emitter (the algal photoreceptor is ~1–2 µm across, scintillons are ~0.5–1.5 µm in diameter) and the size of the entire cell (*Chlamydomonas* is ~10 µm cross, dinoflagellates can be ~150 µm long). Given this, in the simplest model, we consider absorbers and emitters to have a radius *a* ≪ *R*, where *R* is a characteristic size of the cell. Section II sets up the incoming and outgoing problems in mathematical terms, laying out the additional modeling assumptions and definitions. As an example of the problem of interest, the observations of Ueki, *et al*. are analyzed quantitatively to gain insight into the lensing effects that can occur. Analytical results for two-dimensional bodies are presented in Sec. III, including the results of averaging over orientations. Three-dimensional problems are considered analytically in Sec. IV and numerically in Sec. V, where we illustrate strong and complex lensing effects associated with shapes such as those of the genus *Pyrocystis*. In Sec. VI, we discuss possible experiments to examine this problem in greater detail.

## II Preliminaries

We consider two complementary problems, each motivated by considerations of the natural environment.

### The incoming problem: How cell geometry affects light absorption in photosynthesis

**Fig. 3:**
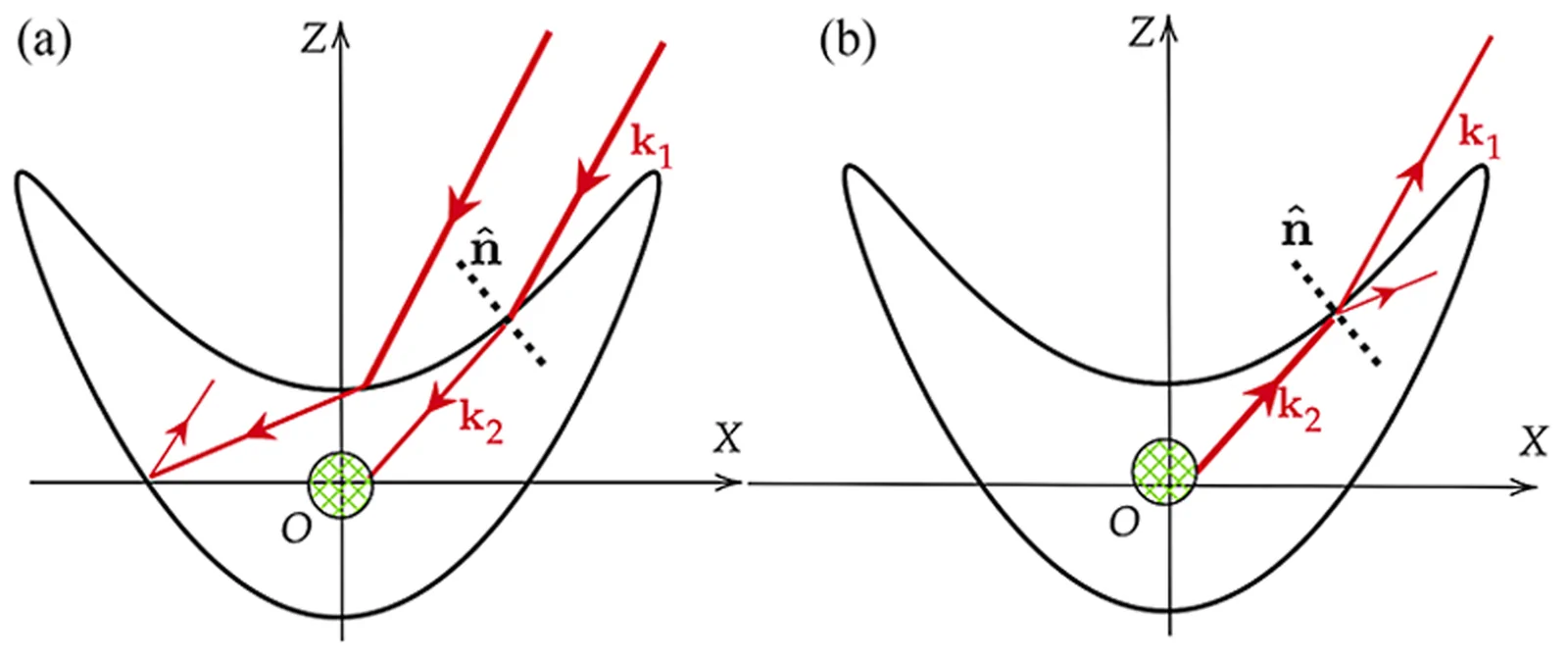
The two optical problems discussed here. Red lines are principal light rays. (a) The incoming problem of how cell shape influences the intensity of photosynthetic light falling on a chloro-plast. (b) The outgoing problem explores how the bioluminescence emission is influenced by the cell geometry.

As shown in Fig. 3(a), this problem explores how a curved cell wall modifies the light intensity at a specific location inside the cell. In the turbulent ocean, microorganisms receive sunlight that is scattered and refracted from nearly all directions, while their random orientations within the flow further homogenize the light distribution. We consider the case in which the cell geometry and random flows are such that there is a uniform angular distribution of cell orientations, so the incoming light appears isotropic from the cell’s perspective. The mathematical problem of interest is then the light intensity received by a small target within the cell relative to that in the absence of the surrounding cell.

### The outgoing problem

*How cell geometry shapes bioluminescence emission*. In marine environments, suspended dinoflagellates respond to the fluid flows associated with ambient turbulence and disturbances from large predators by giving off bioluminescent flashes. Figure 3(b) illustrates the mathematical problem of interest: how a cell’s shape alters the angular distribution of bioluminescent light emitted isotropically from small sources within the cell, undergoing both refraction and internal reflection. Since the cell is much smaller than the distance to predators, the focus is on the direction of emitted light rather than its origin.

Our analysis assumes that the interior of a photosynthetic cell is a homogeneous optical medium with refractive index *n*_cell_, while the surrounding aqueous environment has refractive index *n*_water_ ≃ 1.33. For example, *Chlamydomonas* cells have been reported to exhibit *n*_cell_ ≃ 1.47 in the visible spectrum [2], which implies a relative refractive index, *n* = *n*_cell_*/n*_water_ ≃ 1.1. We adopt this value in our calculations as representative of green algae exposed to light in aqueous environments. Note that the index of refraction of seawater differs by less than 2% from that of fresh water (in the range of 1.33–1.35 [35]), and thus a value of *n* ~ 1.1 is expected to be representative in marine contexts as well.

Although our model assumes a homogeneous refractive index inside the cell, we note that real algal cytoplasm contains membranes, chloroplasts, and scattering elements. For cell sizes much larger than the wavelength of light, geometric optics remains a valid first-order approximation for hotspot positions, even though scattering and absorption may attenuate their intensity. Estimated scattering lengths in algal cytoplasm are typically comparable to or larger than cell dimensions, suggesting that geometric focusing patterns are robust even in the presence of internal heterogeneity.

To model how sunlight enters a cell and contributes to photosynthesis, we apply Snell’s law to incident light rays arriving from the surrounding water,

(1) $$\sin\theta_{i}=n\sin\theta_{t},$$

where *θ*_*i*_ is the angle of incidence in water and *θ*_*t*_ is the angle of transmission inside the cell, both measured with respect to the local normal (see Table I).

The fraction of energy transmitted into the cell quantifies how much unpolarized light passes through the interface. For incident angles *θ*_*i*_ *< θ*_*c*_ = sin^−1^(1*/n*), where total internal reflection does not occur, the Fresnel transmission coefficient *f* (*θ*_*i*_) for unpolarized light is [36]

(2) $$f\left(\theta_{i}\right)=1-\frac{1}{2}\left(\frac{\sin^{2}\left(\theta_{i}-\theta_{t}\right)}{\sin^{2}\left(\theta_{i}+\theta_{t}\right)}+\frac{\tan^{2}\left(\theta_{i}-\theta_{t}\right)}{\tan^{2}\left(\theta_{i}+\theta_{t}\right)}\right),$$

where *θ*_*t*_ = arcsin(sin *θ*_*i*_*/n*) is the transmitted angle determined by Snell’s law. Normal incidence (*θ*_*i*_ = 0) gives the maximum transmittance rate *f* (0) = 4*n/*(*n* + 1)^2^. The quantity 1 − *f* (*θ*_*i*_) is the proportion of light reflected back into the environment; it determines how much light penetrates the cell for internal processes.

We define the *boost factor η* as a measure of light amplification induced by the cell’s geometry. For the outgoing problem, *η* = *d*Ω_2_*/d*Ω_1_ is the ratio of differential solid angles *d*Ω_*i*_ subtended at the internal source and at the exit (far field), capturing how rays from a point source diverge or are focused by the cell as they leave. For the incoming problem, *η = dA*_1_*/dA*_2_ expresses the relative change in projected beam area between an incident patch at the cell boundary and a focal area at the internal absorber, reflecting how geometry concentrates incoming light onto internal targets.

To simplify the analysis of light-ray interactions, we adopt the *chief-ray approximation*, in which all rays are assumed to deviate only slightly from a central principal ray. This reduces geometric complexity, while preserving essential directional and focusing behavior.

### Example: Optical boost at the algal eyespot

**Fig. 4:**
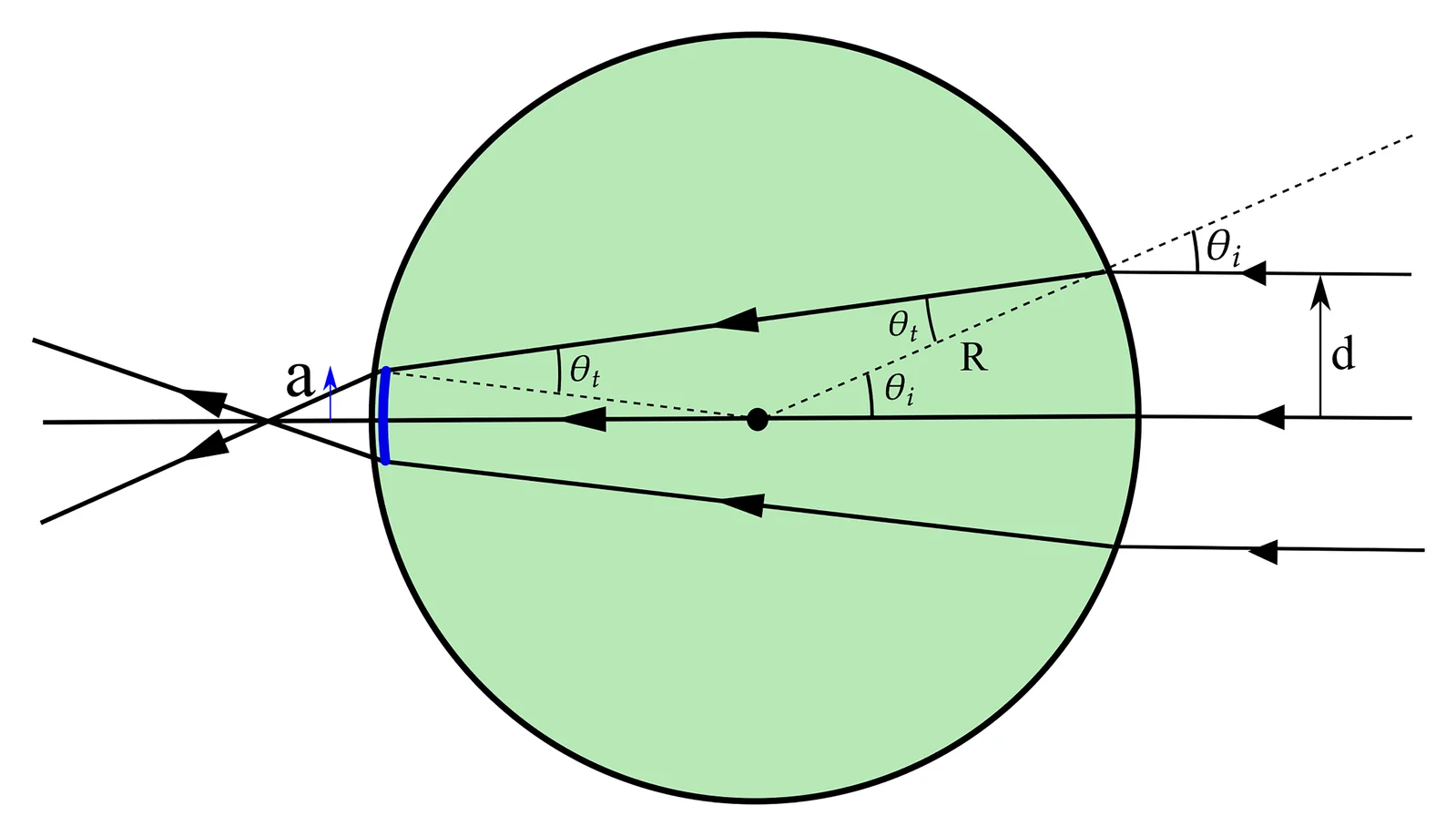
Analysis of the eyespot mutant in *Chlamydomonas*. The photosensor (blue) is located at the inner boundary of the cell. Although incoming light rays focus outside the cell, a gathering effect is experienced by the photosensor.

We begin with a quantitative analysis of the results of Ueki, *et al*. [2] on the *eyeless* mutant of *C. reinhardtii*. Figure 4 shows the geometry; a cell of radius *R* has a photosensor of radius *a* at its periphery, modeled as a circular patch. Light enters the cell from the opposite side, refracts at the surface, and the rays converge past the cell. A cone of these rays hits the photoreceptor. The maximum angular deviation of rays that reaches the photoreceptor defines a limiting incident angle *θ*_*i*_ with “impact parameter” *d* = *R* sin *θ*_*i*_ as in classical scattering theory.

Figure 4 shows the geometry of interest. The vertex angle of the large isosceles triangle is *π* − 2*θ*_*t*_, so if we add all the vertex angles of triangles touching the center, we find *a/R* + *π* − 2*θ*_*t*_ + *θ*_*i*_ = *π* and obtain a relation between the ratio *ϵ* ≡ *a/R* and the incident and refracted angles,

(3) $$\;\epsilon =2\theta_{t}-\theta_{i}≃\frac{2-n}{n}\theta_{i},$$

where the second relation follows from Snell’s law and is valid for *ϵ* ≪ 1, where a small-angle approximation holds.

The optical boost *η* is the ratio of the areas of the incident light cone to that of the photoreceptor patch. In two dimensions, and in the small-angle approximation, this corresponds to the length ratio,

(4) $$\eta_{2\text{D}}=\frac{2d}{2a}≃\frac{\theta_{i}}{\epsilon }=\frac{n}{2-n}.$$

In three dimensions, the incoming rays span a circular disk of radius *d* and the receiving region is a disk of radius *a*, so the boost becomes the area ratio,

(5) $$\eta_{3\text{D}}=\frac{\pi d^{2}}{\pi a^{2}}=\eta_{2\text{D}}^{2}={\left(\frac{n}{2-n}\right)}^{2}\approx 1.49,$$

where we have used the value *n* = 1.1 for *Chlamydomonas*. This remarkably simple result shows that the index of refraction alone determines the boost for a simple spherical geometry. The quantity *η* − 1, the additional flux of light onto the photoreceptor, is

(6) $$\eta_{3\text{D}}-1≃\frac{4(n-1)}{{\left(2-n\right)}^{2}},$$

which is positive only when the relative index *n* > 1.

The large boost in Eq. (5) implies that an eyeless *Chlamydomonas* cell experiences two very distinct signals during each rotation about its body-fixed axis, the one from behind being 50% stronger than the other. Not surprisingly, that significantly larger signal dominates and the cell moves opposite to the wild type. A theoretical analysis of the phototactic dynamics of eyeless mutants is presented elsewhere [37].

## III Analytical Results For 2D Bodies

In this section, we study the incoming problem for 2D bodies, progressing from simple to complex. We scale lengths by the size *R* of the body, so that the small target has radius *ϵ* = *a/R* (similar to the example in Sec. II) and the bundle of light rays that intersects the target has half width *δ* = *d/R*.

### A The circle

**Fig. 5:**
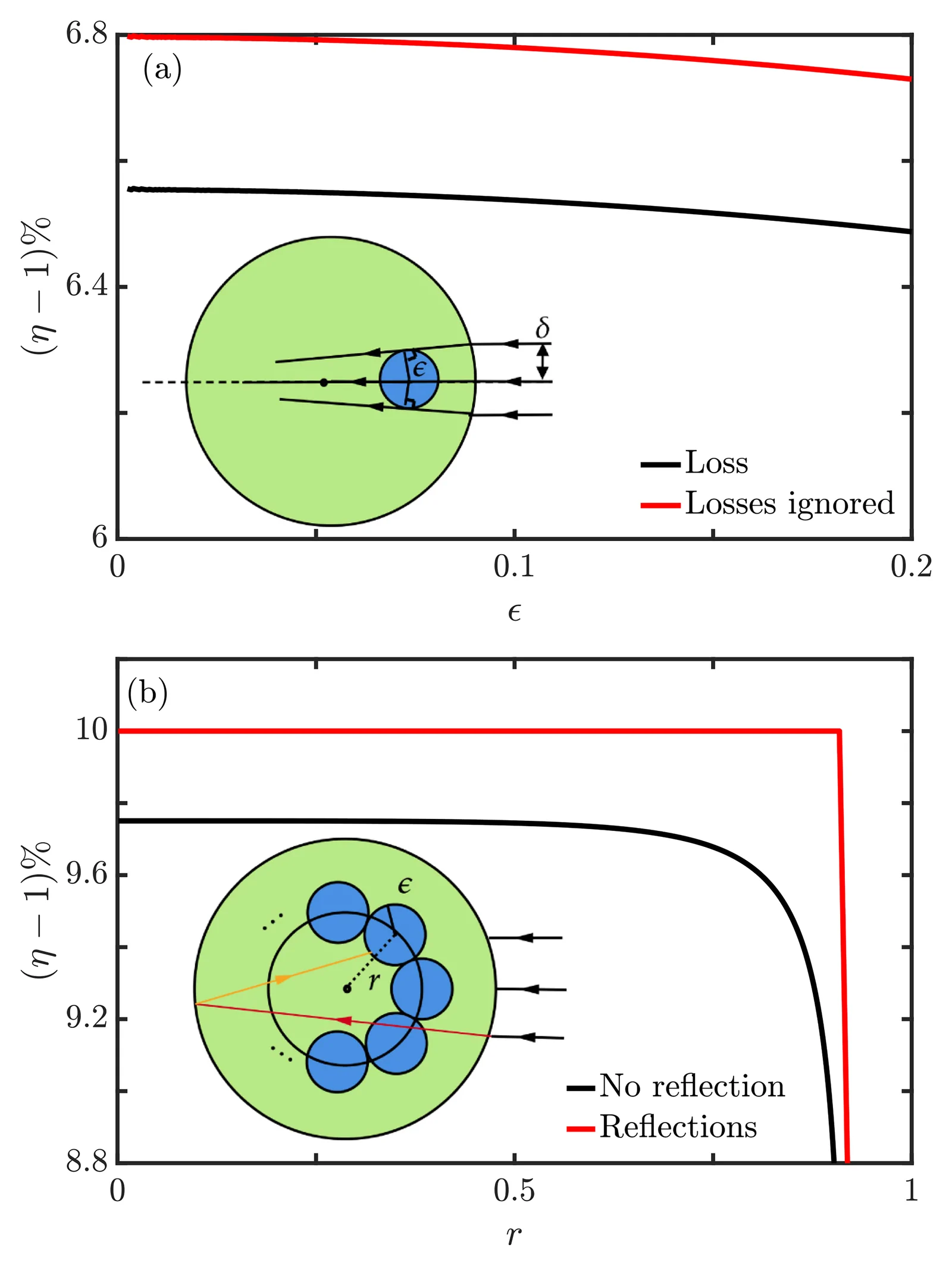
Incoming problem for a circle. (a) Boost vs ball size for *r* = 0.3 and relative index *n* = 1.1. As *ϵ* increases, *η* decreases, indicating weaker lensing and greater losses [Eq. (2)] when incident angle is large. (b) Disks of radius *ϵ* densely populate a circular shell. When multiple internal reflections are included, a ray (red) reaching the second disk is counted via the shell’s absorption. The boost is constant when all reflections (orange line) are included, until the ball reaches the total internal reflection zone where increasing *r* can no longer gain more energy. If reflections are excluded, *η* drops more near the upper boundary due to increased losses.

We begin with the simplest case, a homogeneous circular body, which serves as a baseline for understanding how light refracts and concentrates within 2D bodies. Figure 5(a) shows the setup: A small, fully absorbing test ball of radius *ϵ* is at position (*r,ϕ*) where *r* ∈ [0, 1] is the (scaled) radial distance from the center and *ϕ* is the angle relative to the incoming light direction.

We first consider the case where the test ball is at the center of the circle to evaluate the maximal focusing effect. Because of the circular symmetry, we may take the incoming rays to be parallel and incident from the +*x* direction without loss of generality. The boundary entry points of rays that ultimately graze the edges of the test ball define the incident angular window. If a grazing ray enters at a point on the circle at angle *θ*_*i*_, then, since the normal to the circle at that point is radial, we have *δ* = sin *θ*_*i*_. And as the interior angle between the refracted ray grazing the target and the radial line from the origin that intersects the points of entry is *θ*_*t*_, we have the second relation *ϵ* = sin *θ*_*t*_, and thus *δ* = *ϵn*. Thus, the effective “window” of rays that can reach the test ball spans a lateral width *δ* that increases with the relative refractive index *n*. Note that the distance *δ* ⩽ 1, so the corresponding boost *η*, i.e., the ratio of this width to the width of the test ball, is

(7) $$\eta =\frac{\delta }{\epsilon }=\min\{n,1/\epsilon \}.$$

The boost equals the relative refractive index until it reduces because the ball can receive light with, at most, half width *δ* = 1. The transition also occurs when total internal reflection occurs. This clean result captures the idealized case with perfect transmission and no optical loss, showing that absorption at the center scales linearly with the refractive index *n*. In reality, partial reflection occurs at the boundary, especially for rays striking at oblique angles, due to refractive mismatch with the surrounding medium.

To incorporate such losses, we use the angle-dependent transmission coefficient *f* (*θ*_*i*_) approximated using Eq. (2) for unpolarized light. The net boost factor, corrected for reflection losses, is then

(8) $$\;\eta =\frac{1}{2\epsilon }\int_{-\theta_{\max}}^{\theta_{\max}}d\delta \left(\theta_{i}\right)f\left(\theta_{i}\right)=\frac{1}{\epsilon }\int_{0}^{\theta_{\max}}d\theta_{i}f\left(\theta_{i}\right)\cos\theta_{i},$$

where *θ*_max_ = max {sin^−1^(*nϵ*),*π/*2} is the angular window of rays that refract to the center and cos *θ*_*i*_ is the Jacobian *∂δ*(*θ*_*i*_)*/∂θ*_*i*_. Equation (8) reduces to Eq. (7) when *f* = 1.

### B Effect of absorber size

We now consider test ball locations with *ϕ* = 0, so the geometry remains symmetric about the *x* axis, but with *r >* 0. The boundary entry points of rays that graze the ball define a narrow angular range of incident rays. Using the geometry in Fig. 5(a), the condition for a ray to reach the disk is

(9) $$r\;\sin\left(\theta_{i}-\theta_{t}\right)+\sin\theta_{t}=\epsilon ,$$

which implicitly defines *θ*_*i*_ as a function of *ϵ* and *r*. Once *θ*_*i*_(*ϵ*) is known, the boost factor *η* is calculated from Eq. (8). The resulting boost profile is plotted in Fig. 5(a) for *r* = 0.3. The boost is always smaller than the 10% boost when the ball is placed at the center, as in Eq. (7), because lensing is weaker closer to the light source. In the small-angle limit, Eq. (9) yields the boost

(10) $$\eta ≃\frac{n}{1+r(n-1)},$$

which interpolates between the limit *n/*(2 − *n*) in Eq. (4) for *r* = −1, *η* = *n* for *r* = 0, and *η* → 1 as *r* → 1. The boost profile is continuous and converges to a constant value as *ϵ* → 0. This validates the use of small but finite test balls in simulations to estimate local light intensity. In biological terms, this corresponds to evaluating the light absorption by a small chloroplast placed at a given location within the cell. Because diffraction and coherence effects are negligible at this scale, the ray-based results are expected to agree with those from wave-optics models in the geometric optics limit.

### C Angular averaging

In natural biological contexts, light rays are likely to come from all directions relative to a cell. The results above should then be averaged over the distribution of incoming light rays, and the simplest assumption is a uniform distribution of the angle *ϕ* ∈ [0, 2*π*]. We can compute the boost at each angle and perform this average or, equivalently, we can densely distribute *N* replicas of the test ball within a circular shell at radius *r*, with each ball interacting with light rays from different directions, as illustrated in Fig. 5(b).

The circumference of the shell is the sum of the diameters of all the balls, so we have 2*ϵN* = 2*πr*. The total energy *E*_tot_ absorbed by the shell and the average energy *E*_avg_ absorbed by an individual test ball are related by

(11) $$E_{\text{tot}}=\alpha NE_{\text{avg}},$$

where *α* is the inverse of the averaged number of times a light ray intersects a ball before reaching the cell wall. We will show that the factor *α* = 1*/π* in two ways. First, note that the average energy absorbed is proportional to the circumference, as proved below in Eq. (31), so *E*_tot_*/NE*_avg_ = 2*πr/*2*πϵN*, which confirms the result. Second, if we take the limit *r* → 0 while maintaining *ϵ/r* fixed to be a small number, we are implementing a similarity transformation in which all ratios remain the same, and Eq. (11) still holds, with the same *α*. In this limit, all small balls approach the center, at which the total energy is the linear function *E* (*r*) = 2*rn f* (0), so

(12) $$\frac{E_{\text{avg}}}{E_{\text{tot}}}=\frac{\epsilon }{r},$$

which then yields *α* = 1*/π*.

The light intensity profile as a function of the distance from the center of the circle is shown in Fig. 5(b), using the energy-averaging framework above. At each radial position *r*, the shell can be treated as a disk of radius *r* centered within the cell, and the local boost *η*(*r*) is computed using Eq. (8), with *θ*_max_ =max {sin^−1^(*nr*),*π/*2}. As *r* → 1, the shell approaches the boundary of the circle, and the transmitted light at large *θ*_*i*_ experiences greater loss when crossing the cell wall, as described by Eq. (2). This results in a decrease in *η*(*r*), illustrated by the black curve in Fig. 5(b), which drops sharply beyond *r* ≈ 0.75.

We extend this energy absorption analysis to include an arbitrary number of internal reflections for the test ball. As before, the shell around the central disk is densely populated with smaller disks. These do not absorb light rays directly, as the presence of one does not interfere with another disk intercepting the same ray. Thus, we simply track the number of times a light ray passes through the shell. The total absorbed energy consists of the initial contribution from the ray passing through the cell wall, along with all subsequent internal reflections [see illustration in Fig. 5(b), where the thin red ray represents the first reflection]. By symmetry, each reflected ray follows the same path as the initial one, preserving its angle of incidence.

Denoting the incoming light-ray energy at angle *θ*_*i*_ as *E* (*θ*_*i*_), the total energy absorbed by the shell is

(13) $$\begin{array}{c} E_{\text{tot}}\left(\theta_{i}\right)=E\left(\theta_{i}\right)f\left(\theta_{i}\right)+E\left(\theta_{i}\right)f\left(\theta_{i}\right)\left[1-f\left(\theta_{i}\right)\right]\\ +E\left(\theta_{i}\right)f\left(\theta_{i}\right){\left[1-f\left(\theta_{i}\right)\right]}^{2}+\cdots \\ =\frac{E\left(\theta_{i}\right)f\left(\theta_{i}\right)}{1-\left[1-f\left(\theta_{i}\right)\right]}=E\left(\theta_{i}\right). \end{array}$$

Thus, the contributions from all internal reflections exactly cancel the transmission losses, resulting in a constant boost given by Eq. (7), with *η* = max {*n*, 1*/r*}. This is illustrated by the red curve remaining flat until it drops significantly beyond *r* = 1*/*1.1 in Fig. 5(b).

### D Duality for a circle

In 2D, it can be shown explicitly that the incoming and outgoing problems are equivalent. We first prove this result for a circle without losses [Eq. (2)] and then proceed to the general case.

**Fig. 6:**
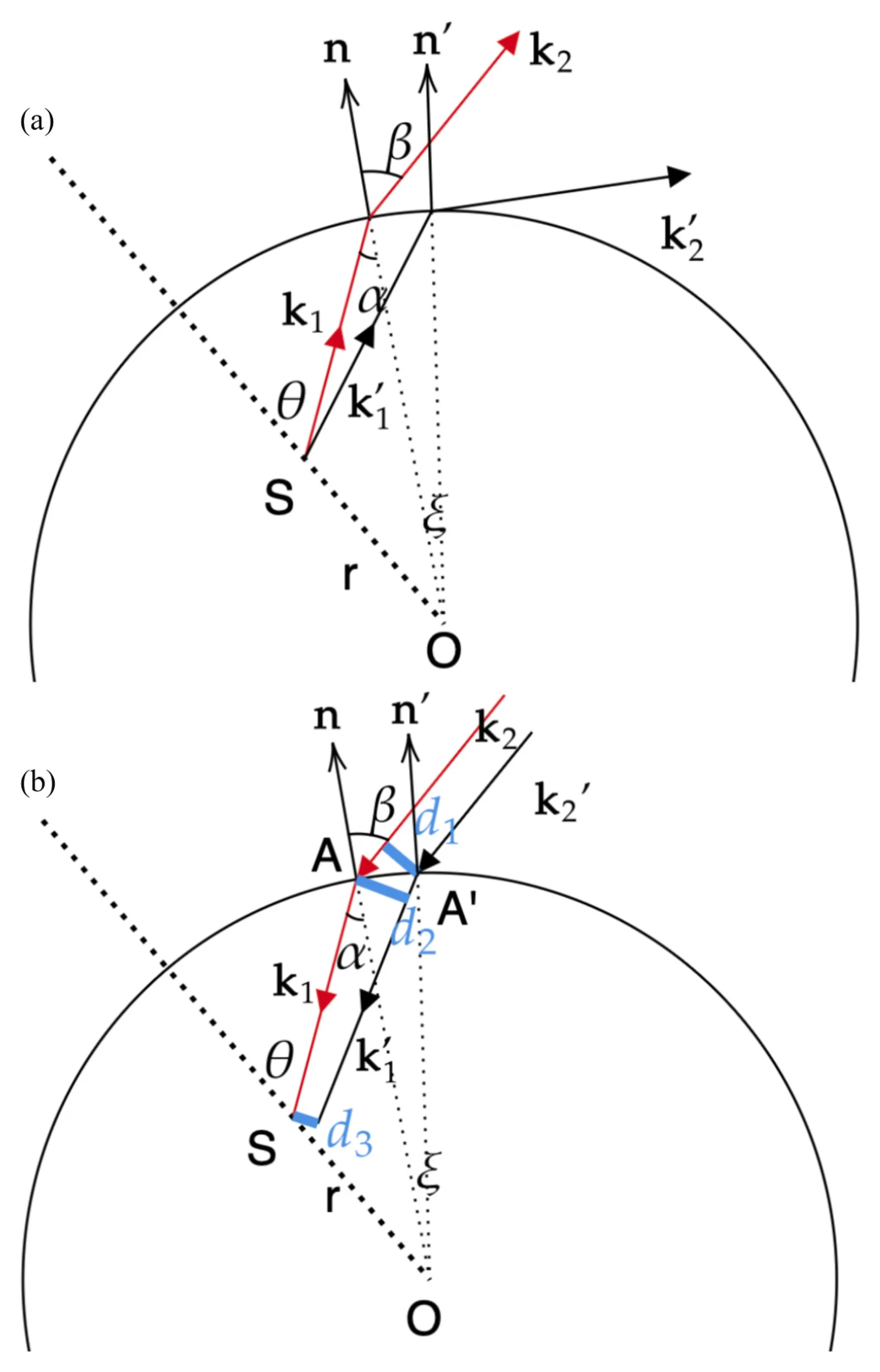
2D geometry to demonstrate duality. (a) Bioluminescence case. Principal light rays are shown in red. We omit the angles between the light rays shown in black, denoted by *θ*′, *α*′, and *β*′. (b) Photosynthesis case. Incoming rays span a length *d*_1_ and the absorber has a projected length *d*_3_.

Consider first the outgoing problem. We adopt the notation ∠(**u, v**) for the angle between vectors **u** and **v**. With reference to Fig. 6(a), the geometric boost for bioluminescence *η*_*B*_ is defined as the limiting ratio of angles,

(14) $$\eta_{B}(\theta )=\underset{∠\left(k_{1},k_{1}^{\prime }\right)\to 0}{\lim}\frac{∠\left(k_{1},k_{1}^{\prime }\right)}{∠\left(k_{2},k_{2}^{\prime }\right)}.$$

With *α* = ∠(**k**_1_, −**n**), *β* = ∠(**k**_2_, **n**), and *θ* = ∠(**k**_1_, **SO**), trigonometry and Snell’s Law, given by Eq. (1), yield

(15) $$\frac{\sin\alpha }{\sin\theta }=r\;\;\;\text{and}\;\;\;\frac{\sin\alpha }{\sin\beta }=\frac{1}{n}.$$

For the primed vectors and angles we have the analogous relations, leading to relations for small changes in the angles,

(16) $$\beta^{\prime }-\beta ≃nr\frac{\cos\;\theta }{\cos\;\beta }\left(\theta^{\prime }-\theta \right)=n\left(\alpha^{\prime }-\alpha \right).$$

Let *γ* = ∠(**k**_2_, **OS**) = *θ* − *α* + *β* and, similarly, define *γ*′. Then, from geometry, we find

(17) $$∠\left(k_{2},k_{2}^{\prime }\right)=\gamma^{\prime }-\gamma =\theta^{\prime }-\theta +\alpha -\alpha^{\prime }+\beta^{\prime }-\beta ,$$

and hence, using Eq. (16), we obtain the boost in Eq. (14),

(18) $$\begin{array}{c} \eta_{B}(\theta )=\frac{\theta^{\prime }-\theta }{\theta^{\prime }-\theta +\alpha^{\prime }-\alpha +\beta^{\prime }-\beta },\\ =\frac{1}{n}{\left(\frac{\sin\alpha \sin(\theta -\alpha )}{\sin\beta \sin\theta \cos\alpha }+\frac{\cos\theta \sin\alpha }{\cos\beta \sin\theta }\right)}^{-1}, \end{array}$$

where, in the last line, we have used Eq. (15) and a product-to-sum trigonometric identity.

For the incoming (photosynthetic) case shown in Fig. 6(b), let point A denote the intersection of the refracted ray **k**_2_ with the cell wall, and similarly for A’. The boost is the ratio of projected areas,

(19) $$\eta_{P}(\theta )=\underset{d_{1}\to 0}{\lim}\frac{d_{1}}{d_{3}},$$

where *d*_1_ is the distance between the light rays **k**_2_ and $k_{2}^{\prime }$, and *d*_2_ is the distance between point A and the ray $k_{1}^{\prime }$. *S*′ is a point on $k_{1}^{\prime }$ with **SS**′ ⊥ **SA**, and *d*_3_ = |**SS**′|. Also, let $k_{1}^{\prime }$ be the distance between *S* and $d_{3}^{\prime }$.

Since *ξ* = ∠(**OA, OA**′) ≪ 1, we have ∠(**n, AA**′) ≃ *π/*2 and |**AA**′| ≃ *ξ*. Geometry then imposes the relations

(20) $$d_{1}=\xi \cos\beta ,\;\;d_{2}=\xi \cos\alpha ,$$

and

(21) $$d_{3}≃d_{3}^{\prime }=\xi \cos\alpha -|SA|∠\left(k_{1},k_{1}^{\prime }\right).$$

To work out the angle $∠\left(k_{1},k_{1}^{\prime }\right)=\xi -\left(\alpha -\alpha^{\prime }\right)$, we apply Snell’s Law, give by Eq. (1) and similar to Eq. (16), and obtain the small angle variation

(22) $$\alpha -\alpha^{\prime }≃\frac{\cos\beta }{n\cos\alpha }\left(\beta -\beta^{\prime }\right).$$

Combining with *ξ* = *β* − *β*′, we find

(23) $$∠\left(k_{1},k_{1}^{\prime }\right)=\xi \left(1-\frac{\cos\beta }{n\cos\alpha }\right),$$

from which we obtain, using Eqs. (20) and (21) in Eq. (19),

(24) $$\eta_{P}(\theta )={\left(\frac{\cos\alpha }{\cos\beta }-\frac{\sin(\theta -\alpha )\sin(\beta -\alpha )}{\sin\theta \sin\beta \cos\alpha \cos\beta }\right)}^{-1}.$$

Using trigonometry sum-to-product and product-to-sum formulas then leads to the duality result,

(25) $$\eta_{P}(\theta )=n\eta_{B}(\theta ),$$

namely, the intensity profiles of the two cases are identical up to a factor of *n*. This is the two-dimensional analog of “étendue” discussed below in Sec. IV.

The outgoing light ray traces the full 2*π* angle,

(26) $$\int_{0}^{2\pi }\frac{1}{\eta_{B}(\theta )}d\theta =2\pi .$$

Then, our definition given by Eq. (14) is *dθ/dγ* = *η*_*B*_(*θ*), leading to conservation of the averaged photosynthesis boost,

(27) $$\begin{array}{c} \eta_{P}^{\text{avg}}=\frac{1}{2\pi }\int_{0}^{2\pi }\eta_{P}[\theta (\gamma )]d\gamma \\ =\frac{1}{2\pi }\int_{0}^{2\pi }\eta_{P}[\theta (\gamma )]\frac{d\theta }{\eta_{B}(\theta )}=n. \end{array}$$

Here, the light rays come at an angle *γ* with **OS**, uniformly distributed in [0, 2*π*].

### E Duality for an arbitrary 2D body

For general shapes, any local region can be approximated as an arc segment with a specific radius of curvature. The argument applies universally for *r >* 1 (when the point lies outside the circle) or *r <* 0 (when the shape is concave). Consequently, the incoming and outgoing problems are locally and globally consistent, and Eq. (25) holds.

Note that for concave shapes, some radii of curvature may be negative, which implies that the local circle corresponding to that curvature lies outside the cell. In these cases, light rays tend to scatter rather than converge. Despite this, a similar analysis can be applied. If the test ball is placed at a distance *r* from the origin and has a small radius *ϵ*, the near-axis approximation holds and the generalization of Eq. (24) is

(28) $$\eta =\frac{d}{\epsilon }\approx {\left[1+r\left(1-\frac{1}{n}\right)\right]}^{-1}<1.$$

This result indicates that the test ball experiences a reduced boost, even before considering light losses.

In the presence of transmission loss, Eq. (26) represents energy conservation and applies generally, provided total internal reflection does not trap energy within the cell indefinitely. By the duality relation given by Eq. (25), Eq. (27) also holds universally: if no light path is confined solely by total reflection, the boost for the incoming problem remains unchanged. However, for structures like a circle, regions near the boundary experience total reflection, leading to a reduced average boost there. Likewise for the incoming problem, if the test ball is too close to the boundary, Eq. (7) leads to the reduced boost seen in Fig. 5(b). This issue is discussed further at the end of Sec. IV B.

### F Averaging for 2D shapes: A surface length law

The baseline analysis of a circular test ball can be extended to arbitrary convex shapes, assuming isotropy in orientation—that is, the shape samples all orientations with equal likelihood. This condition is physically plausible for organisms such as algal cells undergoing slow tumbling or Brownian motion. Many unicellular organisms such as *Chlamydomonas* and *Volvox* [38] exhibit such dynamics due to flagellar motion or ambient fluid fluctuations. These lead to statistical averaging over orientations on timescales relevant for light exposure.

We parametrize such a shape as *r*(*θ*), with unit outward normal **n**(*θ*), and consider illumination from a fixed direction **k**_2_. The projected area is given by

(29) $$P=\frac{1}{2}\int_{0}^{2\pi }d\theta \sqrt{g}\left|\hat{n}(\theta )\cdot k_{2}\right|,$$

where *g* = *r*^2^(*θ*) + (*dr/dθ*)^2^ is the metric factor, and the prefactor of 1*/*2 reflects the fact that only the illuminated half contributes to the projection.

To compute the average projected area over all orientations, we rotate the shape by *θ*_0_ and average over *θ*_0_ ∈ [0, 2*π*].

Under such a rotation, the shape becomes

(30) $$r_{\theta_{0}}(\theta )=r\left(\theta +\theta_{0}\right),\;\;g_{\theta_{0}}(\theta )=g\left(\theta +\theta_{0}\right),$$

and the normal $n_{\theta_{0}}(\theta )$ is **n**(*θ*) rotated by *θ*_0_. Letting **n**′ **n**(*θ*′), the averaged projection becomes

(31) $$\begin{array}{l} \bar{P}=\frac{1}{4\pi }\int_{0}^{2\pi }d\theta^{\prime }\sqrt{g(\theta^{\prime })}\int_{0}^{2\pi }d\theta_{0}\left|\cos\left(\theta_{0}+\cos^{-1}\left|n^{\prime }\cdot k_{2}\right|\right)\right|\\ \;\;\;=\frac{1}{\pi }\int_{0}^{2\pi }d\theta \sqrt{g(\theta )}=\frac{1}{\pi }L, \end{array}$$

where *L* is the perimeter. Notably, the averaged projected area scales linearly with the perimeter and is independent of shape geometry. This reinforces that projection-based absorption for fluctuating 2D convex bodies respects a perimeter-based law.

## IV Analytical Results For 3D Bodies

The simplest 3D shape to analyze is the sphere. For the reference ball at the center, any incident light ray can be analyzed the same way as in 2D. The boost without loss of light due to reflection is analogous to Eq. (7),

(32) $$\eta =\frac{\pi {\left(Rn\right)}^{2}}{\pi R^{2}}=n^{2}.$$

Considering losses, Eq. (8) becomes

(33) $$\eta =\frac{1}{\pi }\int_{0}^{\sin^{-1}\delta }d\theta \int_{0}^{2\pi }d\phi \sin\theta \cos\theta f(\theta ).$$

### A Averaging in 3D: A surface-area law

The same principle discussed in Sec. III E extends to three-dimensional convex bodies with fluctuating orientations. Let the body have a fixed surface area and be parameterized by its radial function *r*(Ω), where Ω denotes a direction on the unit sphere and **n**(Ω) is the outward unit normal. For a fixed direction **k**_2_ of incoming light, the projected area is

(34) $$P=\frac{1}{2}\int d\theta d\phi \sqrt{g}(\theta ,\phi )\left|n(\theta ,\phi )\cdot k_{2}\right|,$$

where the factor 1*/*2 counts the illuminated half of the body, and the metric factor is $\sqrt{g}(\theta ,\phi )=r\sqrt{\left[r^{2}+{\left(dr/d\theta \right)}^{2}\right]\sin^{2}\theta +{\left(dr/d\phi \right)}^{2}}.$

To compute the orientational average of *P*, we integrate over all rotations Ω_0_. Without loss of generality, take **k**_2_ to align with the polar axis and replicate the steps leading to Eq. (31), with *d*Ω_0_ = *dϕ*_0_*dθ*_0_ sin *θ*_0_, yielding

(35) $$\begin{array}{c} \bar{P}=\frac{1}{2}\int \frac{d{\text{Ω}}_{0}}{4\pi }\int \frac{d\text{Ω}}{4\pi }\sqrt{g_{{\text{Ω}}_{0}}}(\text{Ω})\left|n_{{\text{Ω}}_{0}}(\text{Ω})\cdot k_{2}\right|\\ =\frac{1}{2}\int d{\text{Ω}}^{\prime }\sqrt{g}\left({\text{Ω}}^{\prime }\right)\int_{0}^{2\pi }\frac{d\phi_{0}}{2\pi }\int_{0}^{\pi }\frac{d\theta_{0}\sin\theta_{0}}{2}\\ \times \left|\cos\left\{\theta_{0}+\cos^{-1}\left[n(\text{Ω})\cdot k_{2}\right]\right\}\right|=\frac{1}{4}A, \end{array}$$

where *A* is the object’s surface area. As in the 2D case, this result depends only on total surface area. The average projected area of a convex 3D object under uniform orientation fluctuations thus follows a surface-area law.

Also, as in 2D, the above analysis breaks down for concave shapes. In those cases, parts of the surface can shade each other, leading to a reduction in the effective projected area. The impact of self-shadowing must be accounted for separately, as it can significantly alter the absorption characteristics and invalidate the simple surface-area scaling seen in convex geometries. We analyze such effects numerically in Sec. V.

### B Étendue and flux conservation

Étendue (often denoted *ℰ*) can be viewed as the “phasespace volume” of a light beam [39]. It quantifies the spread of light in both position and direction. For a beam passing through a cross-sectional area *A* and contained within a solid angle Ω, the étendue is *E* = *A*Ω. In media where the refractive index varies spatially, this generalizes to

(36) $$ℰ=n^{2}A\text{Ω},$$

incorporating refractive effects on the direction of light rays. *ℰ* is conserved in passive optical systems, as we now show follows from flux considerations.

**Fig. 7:**
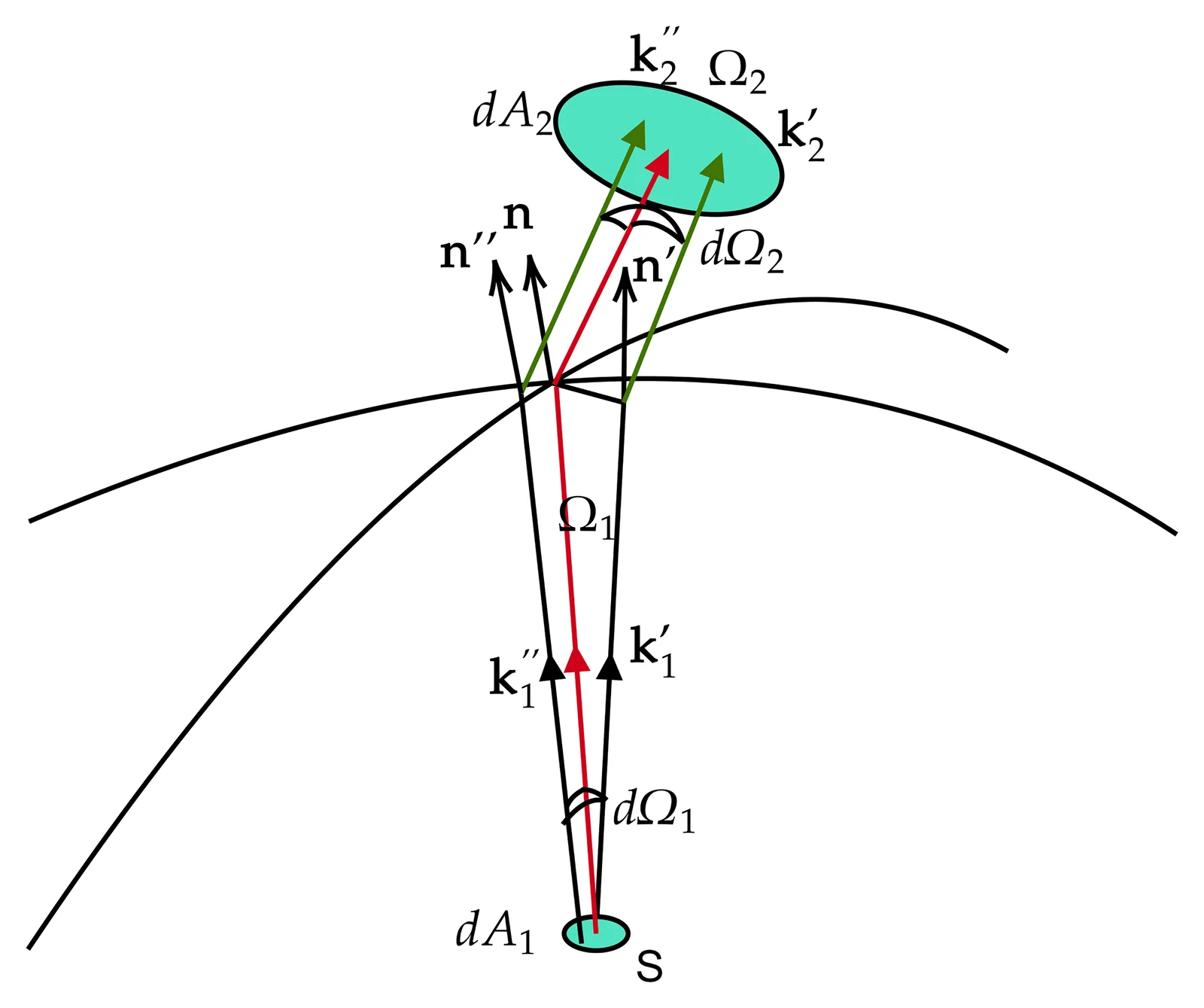
3D geometry of solid angles and projected areas. A test body is located at point *S*, with the cell boundary indicated by two arcs. The principal ray directions Ω_1_ and Ω_2_ correspond to the projected areas *dA*_1_ and *dA*_2_, and to the solid angles *d*Ω_1_ and *d*Ω_2_. The light-ray directions shown represent the outgoing case; for the incoming case, the directions are reversed by ray tracing.

Assume a beam of light crosses the interface without loss and consider a corresponding set of rays defined in medium 1 by an area element *dA*_1_, with chief-ray direction Ω_1_ and solid-angle element *d*Ω_1_. After refraction into medium 2, these same rays will pass through some (generally different) area element *dA*_2_ and subtend a solid angle *d*Ω_2_ with direction Ω_2_ (see Fig. 7). To connect *d*Ω_1_ and *d*Ω_2_, we must understand how a cone of rays in medium 1 maps into medium 2. From the differential of solid angle in spherical coordinates,

(37) dΩ=sinθdθdϕ,

we differentiate Snell’s law and obtain

(38) $$n\cos\theta_{t}d\theta_{t}=\cos\theta_{i}d\theta_{i}.$$

Since reflection does not change the azimuthal angle, we can multiply by *dϕ* on both sides and use Snell’s law again to obtain

(39) $$\cos\theta_{t}d{\text{Ω}}_{2}=\frac{1}{n^{2}}\cos\theta_{i}d{\text{Ω}}_{1}.$$

The light rays intersect the boundary in an area element *dA*, and if we define $dA_{1}^{⊥}=dA\cos\theta_{i}\;$ and $dA_{2}^{⊥}=dA\cos\theta_{t}$, we find

(40) $$\frac{dA_{1}^{⊥}}{dA_{2}^{⊥}}=n^{2}\frac{d{\text{Ω}}_{2}}{d{\text{Ω}}_{1}}.$$

Under the chief-ray approximation, each point in the area element has the same solid angle. The left-hand side of Eq. (40) is recognized as the boost factor for the incoming problem and the right-hand side as that of the outgoing problem. We thus establish that for each solid-angle direction *Ω*_1_ for the outgoing problem,

(41) $$n^{2}\eta_{B}\left({\text{Ω}}_{1}\right)=\eta_{P}({\text{Ω}}_{2})\text{.}$$

where *η*_*B*_ and *η*_*P*_ are boosts in the bioluminescence (outgoing) and photosynthesis (incoming) cases defined in Eq. (14) and Eq. (19). The duality in Eq. (41) is numerically verified in the next section, where the outgoing and incoming configurations yield equivalent intensity distributions.

In the same spirit as Eq. (27), we may calculate the average boost for the photosynthesis problem by integrating over incoming angles,

(42) $$\begin{array}{c} \eta_{P}^{\text{avg}\;}=\frac{1}{4\pi }\int d{\text{Ω}}_{2}\eta_{P}\left({\text{Ω}}_{2}\right)=\frac{1}{4\pi }\int d{\text{Ω}}_{1}\frac{\eta_{P}\left[{\text{Ω}}_{2}\left({\text{Ω}}_{1}\right)\right]}{\eta_{B}\left({\text{Ω}}_{1}\right)}\\ =\frac{1}{4\pi }\int d{\text{Ω}}_{1}n^{2}=n^{2}, \end{array}$$

where, in the second line, we have used *η*_*B*_(Ω_1_) = *∂*Ω_2_*/∂*Ω_1_ as the Jacobian. The average boost is only a function of the relative refractive index *n*, and is irrespective of the concave shell shape and the location of the test ball.

In fact, the argument above works for the case of multiple refractions and when accounting for transmission losses as in Eq. (2). For the incoming problem, a principal light ray hits the test ball after the initial refraction and further reflections; we can trace back the ray from the test ball outside, which becomes the outgoing problem. The conservation of étendue ensures that the duality of the boost holds at any point. The loss at the interface is also the same.

In considering all reflections and loss, let us label the boost by the number of reflections, *i*, that have occurred thus far. The outgoing boost is $\eta_{B}^{i}\left({\text{Ω}}_{1}\right)=f^{i}\left({\text{Ω}}_{1}\right)d{\text{Ω}}_{2}/d{\text{Ω}}_{1}\text{,}$, where $f_{i}\left({\text{Ω}}_{1}\right)={\text{Π}}_{m=1}^{i}\left[1-f\left(\theta_{r}^{m}\right)\right]f\left(\theta_{t}\right)$ accounts for the loss according to Eq. (2). We similarly modify the definition of the incoming boost to account for multiple reflections, including losses. For the outgoing problem, all light will exit the body after a sufficiently large number of reflections, unless the situation is highly symmetric and some light rays are trapped by total internal reflection [see Fig. 10(b) where, near the edge, the average boost reduces to below 1]. Then, energy conservation gives Σ_*i*_ *f* ^*i*^(Ω) = 1, and thus

(43) $$\eta_{B}^{\text{avg}}=\int \frac{d{\text{Ω}}_{1}}{4\pi }\;\sum_{i}\eta_{B}^{i}\left({\text{Ω}}_{1}\right)=\int \frac{d{\text{Ω}}_{2}}{4\pi }\sum_{i}f^{i}\left({\text{Ω}}_{1}\right)=1.$$

Since losses for incoming and outgoing light rays are the same in each direction, Eq. (41) now reads $n^{2}\eta_{B}^{i}\left({\text{Ω}}_{1}\right)=\eta_{P}^{i}\left[{\text{Ω}}_{2}^{i}\left({\text{Ω}}_{1}\right)\right]$. Denote the direction of the outgoing light ray after *i* reflections as ${\text{Ω}}_{2}^{i}({\text{Ω}}_{1})$. Equation (42) becomes

(44) $$\begin{array}{c} \eta_{P}^{\text{avg}\;}=\sum_{i}\int \frac{d{\text{Ω}}_{2}^{i}}{4\pi }\;\eta_{P}^{i}\left({\text{Ω}}_{2}^{i}\right)=\frac{1}{4\pi }\underset{i}{\sum }\int d{\text{Ω}}_{2}^{i}n^{2}\eta_{B}^{i}\left({\text{Ω}}_{1}\right)\\ \;\;=\sum_{i}\frac{1}{4\pi }\int d{\text{Ω}}_{1}n^{2}f^{i}\left({\text{Ω}}_{1}\right)=n^{2}. \end{array}$$

## V Numerical Results For 3D Bodies

In this section, we present numerical results that support the conclusions drawn in the analytical sections above. We consider a two-parameter family of shapes for numerical computations. Built around the parametrization of ellipsoids, these shapes interpolate between the sphere (a suitable model for *Chlamydomonas*) and those with eccentricity approaching unity (appropriate to *P. fusiformis*), and then proceed to ellipsoids bent around their major axis (a shape such as *P. lunula*). In a system of units made dimensionless by the semimajor axis *a* of the ellipsoidal limit, this family can be written as

(45) $$x^{2}+\frac{1}{1-\varepsilon^{2}}\left[y^{2}+{\left(z-\kappa x^{2}\right)}^{2}\right]=1,$$

where $\varepsilon =\sqrt{1-{\left(b/a\right)}^{2}}$ is the eccentricity and *b* is the semiminor axis of the limiting ellipsoids obtained when *κ* = 0. These have major axes lying in the *xz* plane with a radius *r*(*θ, ϕ*) measured from the ellipsoid center of

(46) $$r(\theta ,\phi )=\frac{\sqrt{1-\varepsilon^{2}}}{\sqrt{1-\varepsilon^{2}\;\cos^{2}\;\theta }},$$

while for *κ ≠* 0, the shapes are bent in the *xz* plane around the curve *x* = *κx*^2^for *x* ∈ [−1, 1],

(47) $$z=\kappa x^{2}\pm \sqrt{1-\varepsilon^{2}}\sqrt{1-x^{2}}.$$

**Fig. 8:**
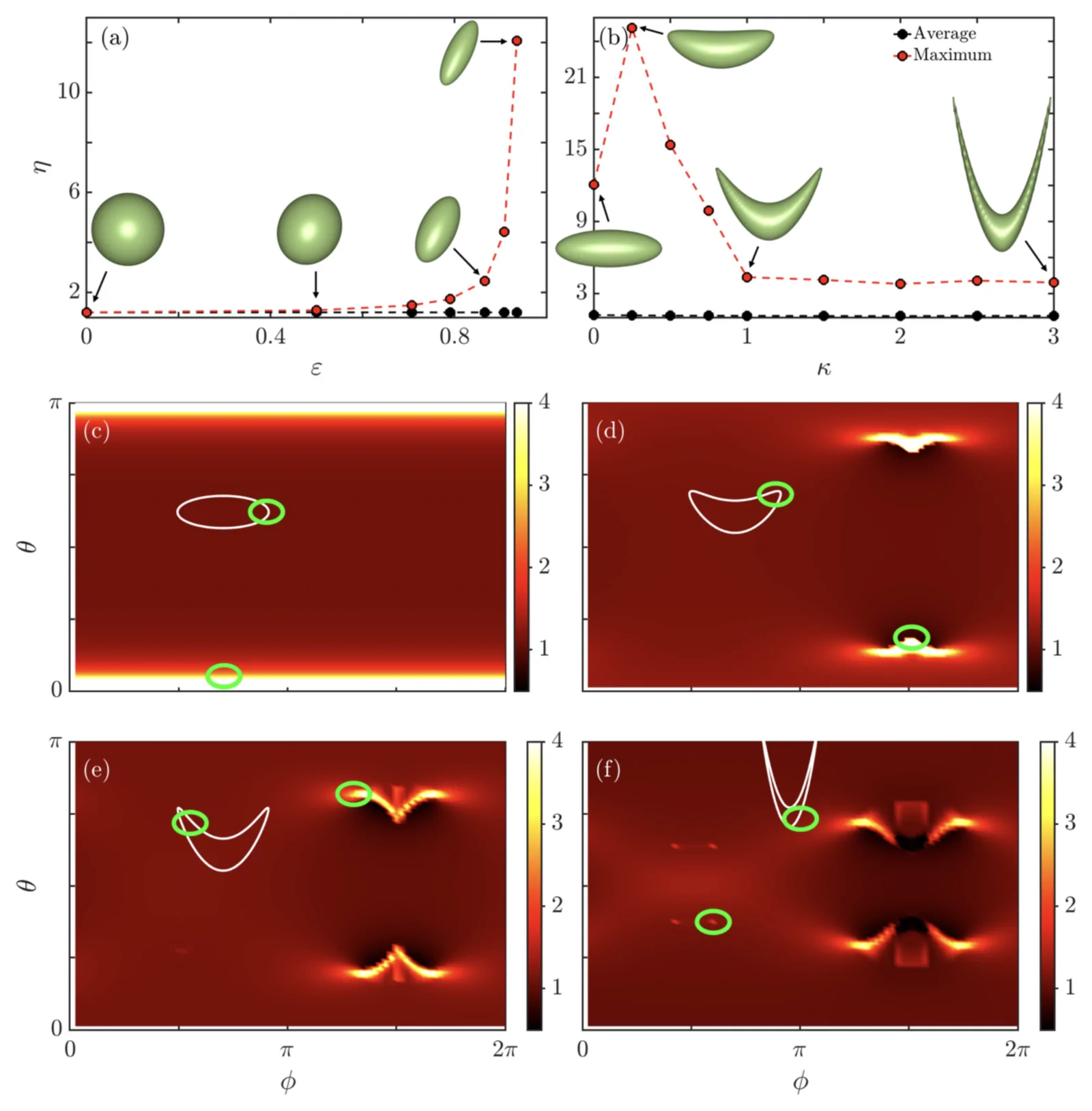
Variation with cell shape of maximum intensity boost at the center of the body. (a) Light intensity observed at the center of ellipses increases as a function of eccentricity *ε*, while the average intensity remains constant. (b) As a function of the curvature parameter *κ*, the maximum boost for bent ellipsoids is nonmonotonic. In (a) and (b), the geometrically accurate renderings of the shapes are included as visual illustrations of the 3D geometry, not as intensity plots. (c)–(f) Intensity boost as a function of angles (*θ, ϕ*), where *θ* denotes the angle between the light ray and the positive *z* axis, and *ϕ* is the azimuthal angle between the projection of the light ray onto the *x* − *y* plane and the positive *x* axis. For an ellipsoid, axisymmetry results in a boost that is independent of *ϕ*. Parameter values are *κ* = 0, 0.5, 1, 3, and eccentricity is $\varepsilon =\sqrt{7/8}$. For (d)–(f) bent shapes, the maximum stays near the direction of the tips. The green circles in (c)–(e) indicate the entry region giving strong boost. Note the focusing of the first reflections circled in green in (f).

Figures 8(a) and 8(b) show such shapes for ranges of *ε* and *κ*.

### A Photosynthesis at the center

To examine how cell shape influences light concentration near the center, we model the chloroplast as a small, perfectly absorbing sphere at the center of the cell, shown as a green circle in Fig. 3(a). Incoming parallel light rays are assumed to arrive uniformly from all directions. We discretize angular directions and emit rays from a plane perpendicular to each specified propagation direction **k**_1_. Here and in following sections, geometric symmetries allow us to restrict the angular sampling to half of the spherical polar angle domains in *ϕ* and *θ*. When the cell is bent, only two planar symmetries remain, resulting in boost-angle profiles with mirror symmetry across *ϕ* = *π/*2 (and 3*π/*2) and *θ* = *π/*2. Both *ϕ* and *θ* are discretized into 40 sampling points. For each direction, 360 000 rays are generated so that the spacing between rays is smaller than the radius of the test ball, while ensuring that the ball remains small relative to the cell geometry.

Computational constraints restrict the region of ray entry to a zone centered on the test ball with a width 15 times its radius—an approximation that remains accurate, as confirmed by the close match between computed intensities in Figs. 8(a) and 8(b) and the analytical prediction given by Eq. (44). Each ray carries equal energy, undergoes refraction at the cell boundary according to Snell’s law given by Eq. (1), and is attenuated by its Fresnel transmission coefficient given by Eq. (2). Each ray undergoes up to 11 reflections and those whose energy drops below 10% of their initial value are discarded to reduce computation time. This setup leads to billions of ray computations for each test ball position. The total absorbed energy is computed and compared with the case without a surrounding cell boundary.

We focus on two metrics: the maximum intensity boost (along the optimal direction) and the average boost (over all directions). In the spherical case, symmetry ensures that *η* is direction independent, so the average and maximum coincide. As the eccentricity *ε* increases, Fig. 8(a) shows that a preferred direction emerges, with the maximum intensity increasing dramatically, from ~1.2 to ~12. Introducing bending with a finite *κ* [Fig. 8(b)] further increases the peak, which exceeds 25 before declining to 4. This nonmonotonic trend arises from the interplay between the increasing distance between the tip and the center, and decreasing radius of curvature radius at the tip. While the initial bending aligns focal zones with the center (by slightly increasing the axis length as initially the center lies between the focus and the tip), enhancing light concentration, further bending misaligns them, decreasing the central boost.

The average intensity is less sensitive to shape. For the sphere and ellipsoids, it remains near *n*^2^ ≃ 1.2 as predicted by Eq. (44). The bending decreases the average slightly, especially near the concave parts of the shell, due to redirection of light rays away from the center as *κ* increases.

### B Angular profile of the boost

To better understand the entry point of the maximum boost and the distribution of light intensity, the boost observed at the center of the body is analyzed as a function of the incoming angles (*θ, ϕ*). For ellipsoids, the boost profile in Fig. 8(c) exhibits the expected axisymmetry in *ϕ*.

For mild bending, Fig. 8(d) shows that the peak region is centered at the tip of the bent shape and is the brightest spot in the profile. As bending increases in Figs. 8(e) and 8(f), light from the tip no longer focuses directly on the center, but rays entering from nearby directions do. This shift in the angular location of intensity peaks is accompanied by a migration of the *θ* center of the bright regions, as the tip curvature becomes sharper.

As the shape becomes more extreme [Fig. 8(f)], additional bright spots and faint striping patterns emerge due to internal reflections of rays within the curved shell. The bright spots correspond to rays that enter the cell, reflect internally at the tip, and are redirected toward the center. The faint stripes are remnants of these multiply reflected paths. These reflection-induced features become especially prominent when the tip region is sharply curved and capable of directing incident rays back inward.

Dark regions in the angular map correspond to incident directions that either miss the central test ball or undergo reflection away from it. These regions typically have a boost near 1, indicating that the light reaching the test ball is neither enhanced nor significantly diminished.

### C Spatial distribution of the boost

**Fig. 9:**
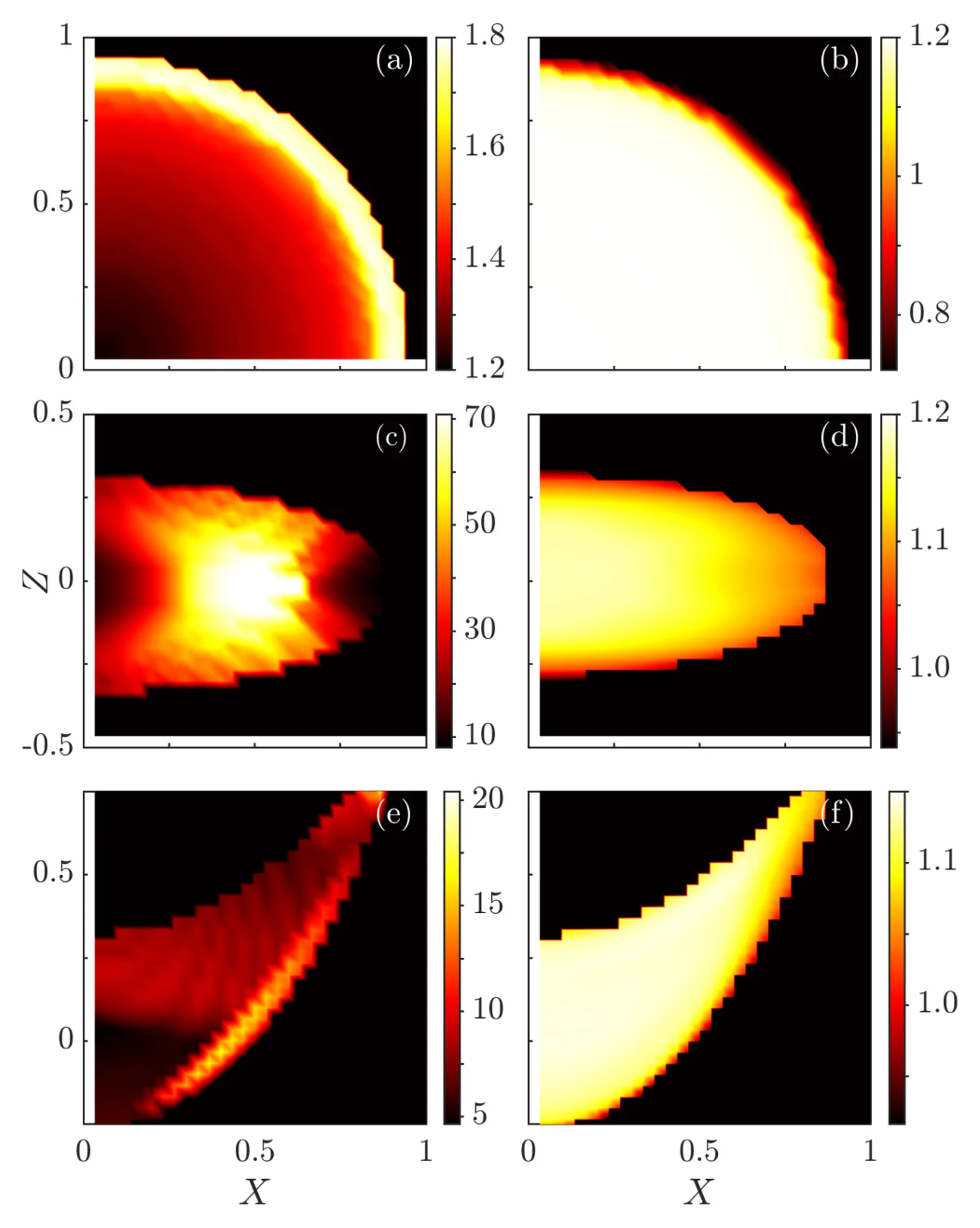
Spatial distribution of intensity boost, showing (a),(c),(e) the average boost and (b),(d),(f) the maximum boost at each location. We use the parametrized surface [Eq. (45)] and take a section at *Y* = 0. *X* and *Z* denote the *x* and *z* coordinates, and *ϵ* and *κ* are defined in Eq. (45). (a),(b) The sphere exhibits a spherically symmetric profile. (c),(d) Ellipsoid with $\varepsilon =\sqrt{7/8}$,(f) *P. lunula* shape with *κ* = 1.

To gain further insight into the light intensity profile—and to explore the potential biological advantages of nonaxisymmetric shapes and organelle distributions—we examine the spatial variation of light intensity within the cell. Specifically, we analyze both the average and the maximum intensity boost at various locations. The results are presented in Fig. 9. All plots consider only a smaller region inside the cell, as edge effects near the boundary make calculations unreliable.

For the sphere, 40 sample points are taken along the radial direction, with the intensity distribution being invariant in the azimuthal angle *ϕ*. Therefore, discretizing the polar angle *θ* into 40 intervals is sufficient. For the ellipsoid, 208 sampling points are distributed inside the cell, leveraging symmetry along the *X* axis. However, due to the reduced symmetry of this geometry, angular profiles are only symmetric about *ϕ* = *π/*2. The high curvature of the ellipsoid surface demands a significantly finer angular grid; coarse sampling results in stripelike artifacts—an issue commonly encountered in raytracing simulations [40]. To eliminate these artifacts in the computed maximum intensity distribution, we discretize the angular space using 160 points in *θ* and 80 in *ϕ*. We use 8100 rays per direction, which has been found to yield accurate results in practice. For the *lunula* example, 387 sample points are used inside the shape, employing the same angular discretization and simulation parameters as for the ellipsoid. However, due to the fourth-order nature of the parametrizing relation [Eq. (45)], the simulation requires substantially more computational time.

Within the sphere, the averaged intensity is highly uniform, with a boost of ~1.2 c as discussed at the end of Sec. IV B and as predicted by Eq. (44), until it drops to *<*0.8 near the boundary. Variations are likely due to numerical errors and the fact that only finitely many reflected rays are considered, supporting the conjecture that all points within a convex shape experience the same average intensity. The maximum intensity is lowest at the center, increasing to ~1.7 near the boundary. As the test ball approaches the boundary, the density profile becomes more uneven due to the proximity to the focal region of light from the antipodal point, as well as the increasing effectiveness of internal reflections. This is larger than the value calculated in Eq. (5) due to consideration of internal reflections.

A similar pattern is observed for the ellipsoidal case. Points near the focal region experience a maximum intensity boost exceeding 70, as light accumulates significantly near the focal point. The averaged intensity remains constant near the center, decreasing slightly from ~1.18 (close to the expected value of *n*^2^) by ~4%, then quickly drops to below 1, again likely due to the limited number of rays considered. For the *lunula* shape, the maximum intensity also shows sensitivity to discretization. However, the maximum boost is generally weaker than in the ellipsoidal case. The thin bright line in Fig.9(e) results from a focusing effect at the tip. The average intensity remains nearly uniform near the center, decreasing from ~1.14 to ~1.1 near the tip due to the concavity of the shape. We also observe a decrease to below 1 near the boundary, similar to the ellipsoid case, likely due to limited light ray regions considered and the energy cutoff of light rays.

These results show that while the average intensity is relatively uniform across shapes, local maxima are strongly influenced by geometry. Focal hot spots, such as those near the ellipsoid focus or the lunula tip, demonstrate how even simple geometric differences can significantly modulate internal light fields, consistent with the directional fluorescence anisotropy measured in elongated diatoms [33].

### D Bioluminescence

**Fig. 10:**
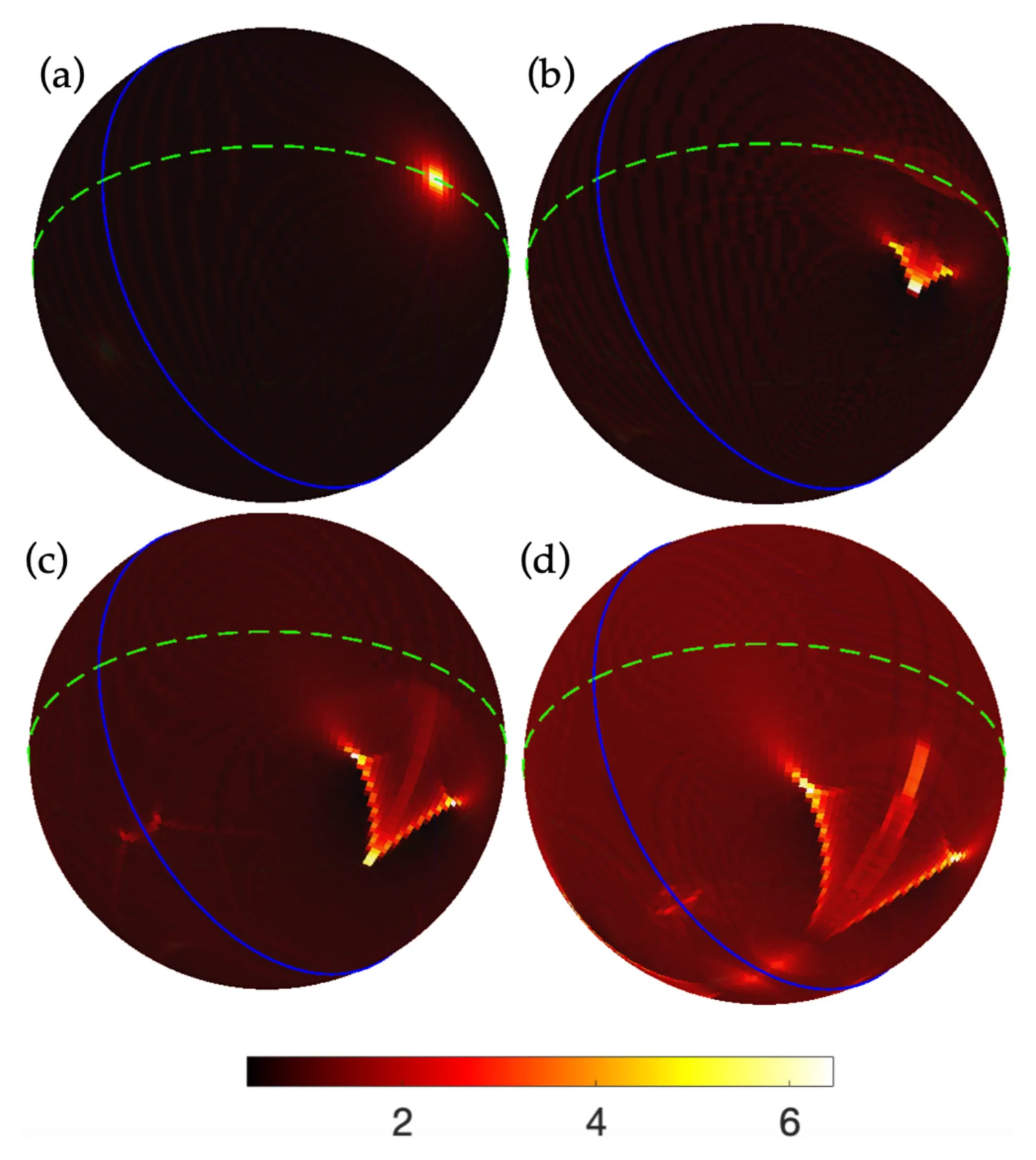
Angular distribution of light emission from the center of a cell. For a source fixed at the center and with eccentricity, $\varepsilon =\sqrt{7/8}$. The bending parameter is *κ* = 0, 0.5, 1, and 2 in (a)– The shapes are plotted in Figs. 8(c)–8(f). The blue solid line marks the *θ* = *π/*2 meridian, while the green dashed line denotes the longitudes *ϕ* = 0 and *π*. The boost region enlarges with *κ*, while the maximum intensity first increases then decreases, a trend also seen in Fig. 8(b). The patterns resemble those of Figs. 8(c)–8(f).

To model the outgoing problem, a point source emits light uniformly from a location inside a given cell shape. Light rays are refracted outward and reflected internally within the cell. Since the cell is small compared to the interorganism distance, the location of outgoing rays can be ignored, and the light intensity distribution is evaluated on an infinite sphere centered at the cell, plotted with respect to the solid angle. The calculation is normalized against the case of a point light source at the center of the observation sphere. The computational process is simplified by generating light rays solely from the test sphere. The spherical grid is discretized into 160 × 160 points in *θ* and *ϕ*, respectively, and 409 600 test light rays are generated from the ball. For reflections, we disregard those carrying less than 1% of their initial energy. The light intensity distribution at each location only requires millions of light-ray realizations and achieves better angular resolution. Future studies on the spatial distribution of photosynthetic boost may benefit from employing bioluminescence-based simulation techniques. The light profile where the test body is placed at the center of various cell shapes is produced in Fig. 10, which shares the same pattern as Figs. 8(c)–8(f). This further confirms the duality in Eq. (41).

**Fig. 11:**
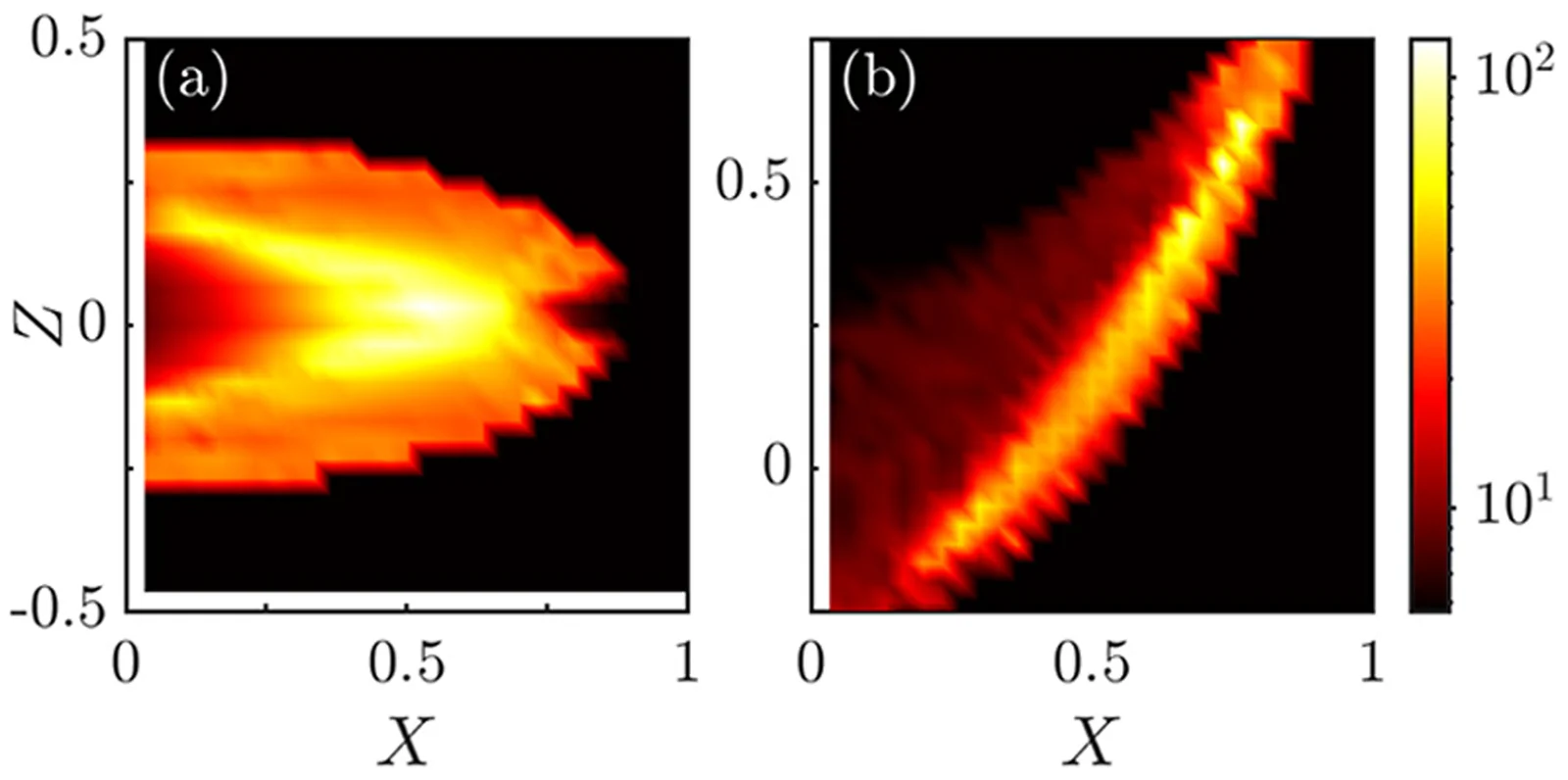
Spatial distribution of maximum emission boost at each location. (a) and (b) correspond to Figs. 9(c) and 9(e). The plot is in logarithmic scale, with bright yellow regions showing strong intensity enhancement.

We similarly examine the spatial distribution of the maximum intensity, as shown in Fig. 11. The maximum intensity values at each point are higher since, at the same angular resolution, the outgoing simulation converges more rapidly to the maximum. Notably, the bright regions remain consistent, in agreement with Eq. (41).

It is worth noting that our simulations of the incoming and outgoing problems handle concave shapes slightly differently. Specifically, light rays that reenter the cell after multiple refractions, or after reflection from the outer surface followed by entry via refraction at a different point, are not included in the analysis. These rays are expected to have negligible impact due to significant energy loss, as described by Eq. (2). Nevertheless, a more refined numerical treatment could be applied for concave geometries such as that of *P. lunula*.

## VI Discussion

We have presented a framework for understanding how cell shape influences the distribution of light in organisms. Our work covers both incoming light, relevant for photosynthesis, and outgoing light, relevant for bioluminescence, and is based on geometrical optics. We introduced the notion of a boost factor *η* to quantify the focusing or defocusing of light within a cell. For simple geometries, such as the circle and the sphere, we obtained exact analytical results. In more complex shapes, such as ellipses and bent geometries motivated by real dinoflagellates, we computed the intensity distributions numerically. In all cases, we find that geometric focusing alone can produce significant spatial variation in the light field, even in the absence of any specialized optical structures.

Transparent, weakly refracting cells can therefore experience substantial internal intensity gradients purely from geometry, with bent cells concentrating light near the outer curvature by more than an order of magnitude, while convex geometries show average boosts close to that of the circle or sphere, indicating that geometry redistributes rather than amplifies total light.

A key observation is that incoming and outgoing problems are related by a geometric duality, derived from conservation of étendue in a passive optical system [39]. Duality allows us to deduce the intensity distribution in one case from that in the other. The duality relation was verified numerically across a range of geometries, and we showed that both configurations yield equivalent angular profiles after appropriate transformation. Biologically, this implies that spatial regions of enhanced light absorption are also potential hotspots for light emission. Observations of chloroplast and scintillon positioning in species such as *Chlamydomonas, Volvox*, and *Pyrocystis fusiformis* suggest that organelles often localize near such predicted focal regions, supporting the functional significance of this geometric duality [26,41,42]. Such organelle localization may be particularly important for microorganism photosynthesis under the low-light conditions found in the Arctic [43] and Antarctic [44].

These results suggest that cells can passively manipulate light intensity using shape alone, a strategy relevant to both photosynthetic light capture and directional signaling. For photosynthetic cells, strategic localization of chloroplasts near focal zones (e.g., tips or curved regions) could maximize light absorption [45,46]. Recent work on both plants [47] and dinoflagellates [48] has revealed details of organelle movements within cells in response to changing light levels.

For bioluminescent organisms, shaping the cell to direct emission could enhance signal directionality. In the physiologically relevant low-index case (*n* ≈ 1.1; Fig. 2), our ray tracing shows that *P. fusiformis* preferentially beams along its long axis, in agreement with recent diatom fluorescence measurements [33], and *P. lunula* displays a left-right intensity imbalance, consistent with geometry-driven leakage rather than high-contrast refraction. These anisotropies could influence signal projection, enhancing visibility in preferred directions while reducing the detection risk in others. Directional focusing is thus functionally relevant for communication and defense: reducing metabolic cost compared to isotropic emission [30].

Combining these directionalities with the tumbling dynamics of elongated objects in strong shear flows, cells may act as “stochastic beacons” of light, producing nontrivial fluctuation statistics of light production in large groups of organisms even though orientation distributions become more isotropic under strong turbulence. Importantly, in low-turbulence environments, elongated phytoplankton such as *Ceratium* and *Lingulodinium* have been shown to align their long axis with persistent shear flows [49,50]. This stabilizes orientation and makes directional bioluminescence ecologically meaningful in such settings. Recent work on diatoms suggests that the existence of light-mediated communication between cells can drive coupled orientational dynamics [51,52] in laminar flow. There may well be implications of cell lensing for both phototaxis and photosynthesis in dense populations of organisms, as evidenced by collective dynamics and instabilities observed in recent experiments [53,54].

Recent studies of dinoflagellate light production and its cellular mechanisms suggest that geometric effects may also influence the triggering process leading to bioluminescent flashes [32]. Tools such as high-speed imaging or optogenetic reporters may provide ways to probe the internal light distribution *in vivo* [55]. Our findings may help explain why certain phytoplankton have evolved strongly eccentric or bent geometries, and they offer a testable hypothesis for subcellular organization driven by optical advantages.

Our model assumes geometrical optics and a homogeneous refractive index inside the cell. These assumptions are valid for cells much larger than the wavelength of light, but effects such as diffraction or interference may become relevant for smaller cells or near high-curvature regions. The presence of strongly absorbing components such as pigments or organelles could also modify the results; the inclusion of absorption is an important future extension. Likewise, time-dependent effects such as rotation or deformation can shift the positions of optical hotspots. This dynamical modulation of the internal light field may allow cells to optimize absorption or emission in response to changing light conditions [16,46]. Time-dependent adaptation processes within cells [56,57] are known to produce complex phototactic responses, including oscillations [58], which may relate to the underlying photosynthetic dynamics [59]; the effects of lensing on these processes have not yet been studied. We also restricted our attention to passive optical structures, neglecting any active light-guiding mechanisms or structural features. In some species, there is evidence of microlenses, reflective layers, or cellular-scale waveguides [60], which could be treated by extending the present framework to include multiple layers or graded refractive index profiles. Coupling the boost factor to light-activated processes such as chloroplast migration or flash triggering may help explain how phototactic and bioluminescent behaviors are coordinated in organisms that rely on both. Finally, it may be possible to study lensing effects in nonbiological systems that display phototactic behavior, such as certain colloidal systems [61].

## Figures and Tables

**Table I:** List of variables and their definitions.


Definition	Variable
Incident and outgoing light rays	**k**_1_, **k**_2_
Incident angle	*θ_i_*
Transmitted angle	*θ_t_*
Reflected angle	*θ_r_* = *θ_i_*
Energy transmission, Eq. (2)	*f*
Intensity boost	*η*
Local surface normal	**n**
Local radius of curvature	*R*
Relative refractive index, Eq. (1)	*n*


## Data Availability

The data that support the findings of this article are openly available [62].
